# Open questions on carbon-based molecules in space

**DOI:** 10.1038/s42004-022-00714-3

**Published:** 2022-08-10

**Authors:** Christopher S. Hansen, Els Peeters, Jan Cami, Timothy W. Schmidt

**Affiliations:** 1 School of Chemistry, UNSW, Sydney, NSW 2052 Australia; 2 Department of Physics & Astronomy, University of Western Ontario, London, ON N6A 3K7 Canada; 3 Institute for Earth and Space Exploration, University of Western Ontario, London, ON N6A 3K7 Canada; 4 SETI Institute, 189 Bernardo Avenue, Suite 100, Mountain View, CA 94043 USA

**Keywords:** Physical chemistry, Organic chemistry, Astrochemistry

## Abstract

It has been a great joint achievement of astronomy, laboratory spectroscopy and quantum chemistry to identify interstellar molecules in various astronomical environments and piece together their origins story from the fragmented evidence. Here the authors provide a sketch of what we know and motivate the asking of open questions on carbon-based molecules in space.

## Alpha and omega: molecules in circumstellar shells and star-forming regions

**Fig. 1:**
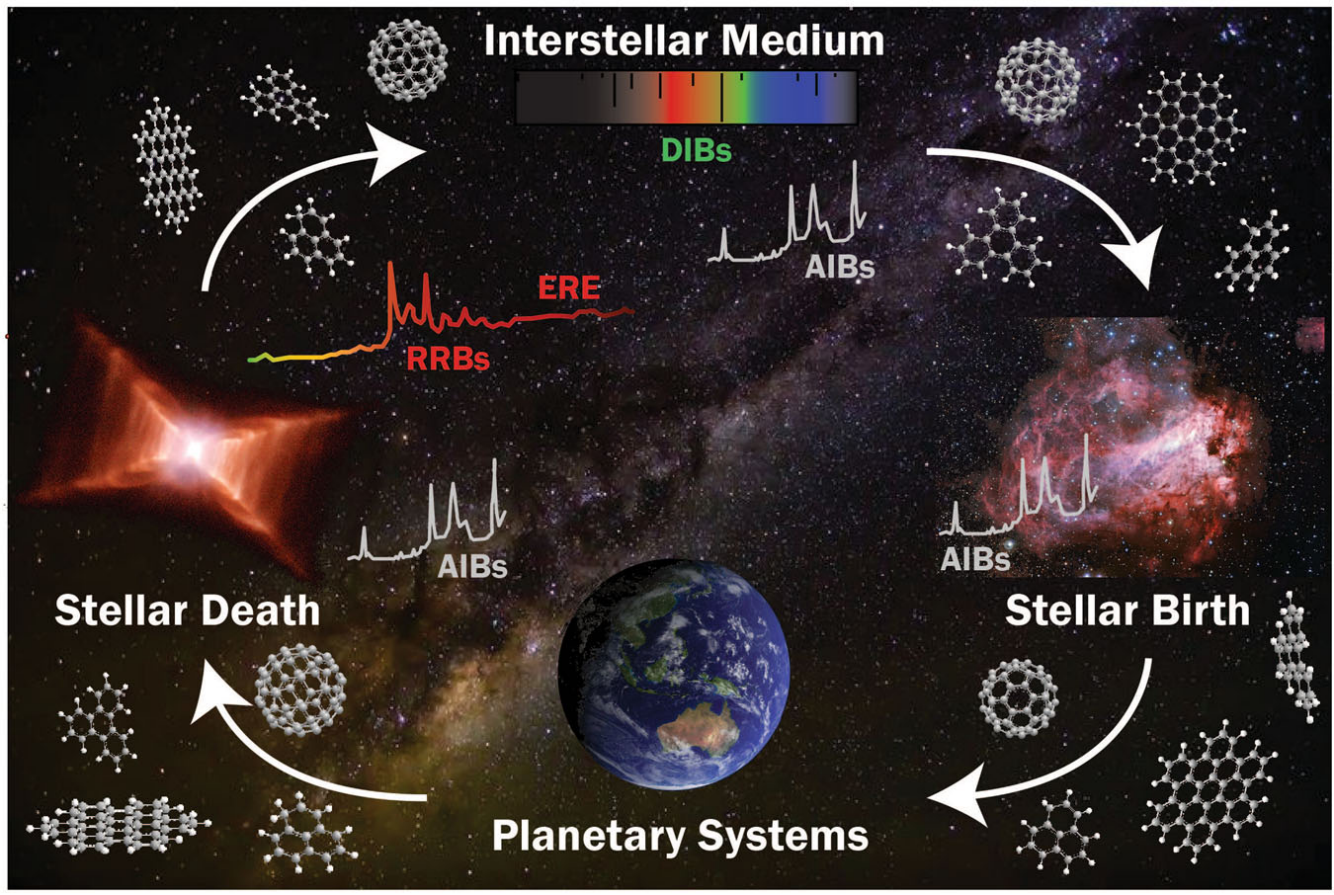
The life-cycle of carbon and associated spectroscopic features. Carbon is cycled between planetary systems and the interstellar medium as stars die and are reborn. Along the way carbon-based molecules exhibit many spectroscopic phenomena posing unanswered questions. AIBs aromatic infrared bands, RRBs Red Rectangle bands, ERE extended red emission, DIBs diffuse interstellar bands. [Red Rectangle Image (left): NASA; ESA; Hans Van Winckel (Catholic University of Leuven, Belgium); and Martin Cohen (University of California, Berkeley). Messer 17 Image (right): European Southern Observatory. Image of Earth (bottom): NASA. Image of Milky Way (dark emu, background): Rowen McRae].

The story of carbon in space starts in the heart of evolved stars, and ultimately results in the complex chemical networks on planetary surfaces which includes life itself. On its journey, carbon reveals itself to earthbound observers through spectroscopic absorptions and emissions across the electromagnetic spectrum (Fig. 1).

When low and intermediate mass stars reach the final stages of their evolution, their pulsating atmospheres swell as they evolve into red giants and lose their outer layers at an ever-increasing pace. Depending on the relative abundance of C and O in their atmospheres, they are classified as C-rich or O-rich. Carbon stars generate a veritable menagerie of molecules, most of which have been identified by their infrared and millimetre-wave absorptions and emissions. The list includes a variety of heteroatoms and functional groups, but the vast majority are carbon-based. These identifications are biased towards molecules with large permanent dipole moments, and as such many long carbon chains have been identified, including cyanopentaacetylene, HC_11_N^1^. Similar species are also observed in stellar nurseries, such as the Taurus molecular cloud. Many canonical organic molecules such as benzene have no permanent dipole moment and must be observed by infrared or electronic spectroscopy. Indeed, after benzene^2^ a second aromatic species was not conclusively identified until 2018 with the observation of cyanobenzene^3^. Shortly after this identification, the two isomers of cyanonaphthalene^4^ were identified in the Taurus molecular cloud and the first pure polycyclic aromatic hydrocarbon was identified in the form of indene^5,6^.

## The aromatic infrared bands

With the advent of infrared astronomy, it became apparent that many astronomical objects glow brightly at certain wavelengths, notably 3.3, 6.2, 7.7, 8.6 and 11.3 μm. This set of bands, originally called the unidentified infrared bands (UIR bands), were identified with polycyclic aromatic hydrocarbons (PAHs)^7^ and their derivatives. Here PAH is taken to mean any molecule substantially described by a polycyclic aromatic hydrocarbon core. The exact nature of these PAHs remains elusive, and until 2021, no specific PAH had been conclusively identified in Space^4^.

PAHs absorb high-energy vacuum-ultraviolet (VUV) photons generated by hot, young massive stars and cool down by radiating infrared (IR) photons, cascading down vibrational quantum states. These aromatic infrared bands (AIBs) comprise up to 15% of the total energy emitted by galaxies and their carriers contain ~20% of all cosmic carbon^8^. However, as these emission features are characteristic of molecular vibrational modes, they are common to all PAH molecules. Inferences must be made based on the variation of relative band intensities, profiles and positions.

The detailed shape and relative intensities of the AIBs are controlled by the composition of the PAH population regarding size, structure, hydrogen coverage, heteroatom substitution and charge state. What is quite certain at this point is that a large proportion of the PAH cohort is singly ionised, and that they are fairly large – comprising 30 or more carbon atoms. Clusters are also thought to contribute to the AIB emission, especially further away from sources of energetic radiation^9^.

## Fullerenes

A PAH wrapped around itself with no edges (and so no hydrogen) is a fullerene, a class of pure carbon clusters first identified in the mid-1980s in experiments attempting to create astrophysically relevant molecules. In 2010, Cami et al. reported the first observations of circumstellar C_60_ and C_70_ by their infrared emission spectra in a young planetary nebula^10^. They have now been observed in star-forming regions, young stars, evolved stars, pre-planetary nebulae, planetary nebulae and the interstellar medium (ISM). In the early 1990s they were posited to carry some near-infrared absorption features in the ISM^11^.

## The diffuse interstellar bands

The ISM exhibits a family of absorption features, collectively known as the diffuse interstellar bands (DIBs). These are widely presumed to be carried by gas-phase, carbonaceous molecules in the ISM. The >500 confirmed DIBs^12^ span the near ultraviolet, visible and near infrared regions of the spectrum, but until recently, no precise carrier was known.

The possible fate of stellar fullerene molecules was revealed in 2015: After 13 years, the project begun in Basel, in 2002, came to fruition, revealing C_60_^+^ to be the carrier of a few DIBs in the near infrared^13^.

## The known knowns

The state of play is that we know there is a great variety of carbon-based molecules present in the molecule-rich environments at the point of stellar death, and in which new star and planetary systems form. The infrared emission indicates that a large part of the inventory is aromatic, including fullerenes. The need of a chromophore to absorb ultraviolet light to trigger AIB emission means that only aliphatic groups bound to aromatic moieties contribute to the AIBs. Nevertheless, absorption spectra towards the galactic centre indicate that around a quarter of the interstellar carbon is aliphatic, and about a third can be attributed to gas phase atoms and small molecules. The rest of the story remains to be elucidated.

## The open questions

### The exact nature of the AIB carriers

The AIBs vary between different sight-lines due to the prevailing photophysical conditions. Hence, they encode a vast amount of information about the physical and chemical environments in which they reside, shedding light on astrophysical processes such as star and planet formation, and galaxy evolution. The best observations to date of PAH sources yield spectra averaged over regions with extremely different properties, thus greatly confusing their interpretation, but knowledge of PAH spectroscopy is also found wanting.

The comprehensive understanding of PAH photochemistry and emission spectra is lacking due to a paucity of comparable laboratory data. Only a few PAHs have been observed in the laboratory by their infrared emission, with poorly characterised internal energy, and none are of the size considered necessary to persist in the ISM^14,15^. Therefore, despite the relevant photophysical process being infrared fluorescence, knowledge of PAH infrared spectroscopy is largely based on their absorption spectra, which are compared to the best available theoretical predictions. Furthermore, for all but the smallest PAHs the calculations do not account for anharmonic effects. And yet, while it is widely accepted that a large part of the AIB emission is due to PAH cations, there is a paucity of infrared spectra of large, gas-phase PAH cations. Furthermore, closed-shell cations—which could represent a large proportion of those observed astronomically—are largely ignored.

What is required is a rigorous union between laboratory emission spectra of large PAHs, and quantum chemical theory that is of high enough quality to accurately predict the band positions, intensities, and profiles as a function of internal energy. This is a great challenge to laboratory astrophysics, but it is one that must be met to thoroughly interpret the AIBs and exploit the information that they encode. This is especially urgent as we enter the James Webb Space Telescope (JWST) era, which will bring forth astronomical data of unparalleled resolution.

### The carriers of the DIBs

PAHs are evidenced by their emission spectra in stellar death and stellar birth. However, little is known of their journey through the ISM between these events. Yet, the interstellar absorption spectrum in the visible region is replete with the spectra of unidentified molecules—the Diffuse Interstellar Bands. Are these features due to highly processed PAHs, or closed-shell cations?

The observation of C_60_^+^ implies that large aromatic molecules are quite likely to be found in the ISM in an ionised state. But, open-shell PAH cations, which result from closed-shell neutral PAHs (e.g., naphthalenium), can be ruled out as carriers of visible DIBs, due to their first excited states lying at low energy: Absorptions to higher excited states in the visible region result in broad spectra due to vibronic coupling, and the DIB features are relatively sharp (they are diffuse only compared to atomic features). However, closed-shell cations resulting from ionisation of resonance-stabilised radicals (e.g., phenalenylium), will exhibit sharp absorption spectra in the visible region.

Electronic spectra of these species are required to rule them out (or in!) as carriers of the DIBs. The challenge, as always, is that there is an intractable multitude of PAHs of the size thought to be relevant, and the carriers might comprise heteroatoms.

### The carriers of the extended red emission and Red Rectangle Bands

The Red Rectangle is an enigmatic pre-planetary nebula which exhibits emission features plausibly aligning with DIBs^16^. It also exhibits a mysterious extended red emission (ERE). One proposal put forward is that the ERE is due to closed-shell PAH dimers. These are dimers of a closed shell neutral PAH clustered with a closed shell PAH cation. This has never been experimentally tested.

### The 217.5 nm extinction hump

This feature is seen throughout the Milky Way, and in other galaxies. The central wavelength is constant along various lines of sight, but the width varies by up to a factor of two. Discovered in 1964, its origin has been speculated upon ever since. Most proposals involve planar, sp^2^ carbon. In the past few years it has been noted that hydrogenated amorphous carbon (HAC) nanoparticles reproduce the hump, presumably due to aromatic domains^17^. In 2010 Steglich et al. created PAH mixtures by laser pyrolysis of gaseous hydrocarbon. They advocated that the hump was due to free-flying PAHs of sizes around 50–60 C atoms^18^.

As of 2017 the hump was attributed to “large PAH molecules or ultrasmall carbonaceous grains”^19^, and the hump was convincingly reproduced in “laboratory-produced polyaromatic disordered carbon grains”^20^.

With the identification of aromatic molecules in space and C_60_ in neutral and cationic forms, the existence of PAHs in the ISM appears more secure than ever. But is the hump due to isolated gas-phase PAHs or clusters? How big do the PAHs need to be to account for the hump, and how much of the interstellar carbon budget would this require?

### Formation and destruction mechanisms

In addition to the open questions on the carriers of astronomical spectroscopic features, there remain questions as to how these molecules are formed, and how they are destroyed. A full picture of carbon-based molecules in space will emerge as we understand what is there and how it came to be there: We need to identify the carriers of the various astronomical absorption/emission features, and understand how the carriers are interrelated. This includes how large molecules are related to the available small molecule building blocks, and how they are processed in various astronomical environments. While the carriers of some of the features mentioned may not ultimately inform us on the broader questions of carbon-based molecules in space, that we cannot identify their carriers is emblematic of our lack of detailed knowledge of the spectroscopy and chemistry of relevant molecules.

## Summary and outlook

A large part of the story of molecular carbon in space is one of aromatic molecules. They dominate the emission in the infrared region of the spectrum and are starting to be identified by millimetre-wave astronomy. The full story will require models to predict the formation and destruction of these species, including their fate in the interstellar medium and the identity of the DIB carriers. However, the models cannot be validated until the carriers of spectroscopic features are conclusively identified.

With the advent of sophisticated ion trapping techniques, it is hoped that the DIB carriers will reveal themselves over the next 5–10 years. A number of research groups are now trapping exotic cations in cryogenic ion traps and extracting nearly unperturbed spectra by helium-atom-tagging. Action spectra obtained from ion mobility spectrometry–mass spectrometry may also prove illuminating. It is certain that more astronomical species will be identified by millimetre-wave astronomy, combined with laboratory chirped-pulse microwave spectroscopy. But, the low-hanging fruit there remains small, neutral species.

To fully understand the wealth of astrophysical information to be provided by the JWST, we require a renaissance in the infrared emission spectroscopy of PAHs. Ideally, PAH cations and neutrals with well-characterised internal energy would be excited by VUV light and their full infrared emission spectra recorded as a function of time. Such an experiment would be of immense value to the astronomical community but pushes laboratory capabilities to their limits. These measurements must walk hand-in-hand with high-fidelity anharmonic quantum chemical calculations, using the best available methods to accurately predict the emission spectra.
